# Steam Efficiently Enhancing CO_2_ Direct Mineralization Steel Slag Towards Actual Production: Phase Evolution, Microstructure, and Mechanisms

**DOI:** 10.3390/ma18204786

**Published:** 2025-10-20

**Authors:** Xiaoqian Wang, Changsheng Yue, Guanghua Lu, Xiangtao Huo, Guilan Yi, Haokun Li, Min Guo, Mei Zhang

**Affiliations:** 1 State Key Laboratory of Advanced Metallurgy, School of Metallurgical and Ecological Engineering, University of Science and Technology Beijing, Beijing 100083, China; 2 Energy Conservation and Environment Protection Co., Ltd., MCC Group, Beijing 100088, China; 3 National School of Elite Engineering, University of Science and Technology Beijing, Beijing 100083, China; 4 Technology Center, Taiyuan Iron and Steel (Group) Co., Ltd., Taiyuan 030003, China

**Keywords:** steel slag, direct carbonation, steam-promoted mechanism, steam-enhanced carbonation

## Abstract

About 120 million tons of steel slag are produced annually in China, making it one of the largest sources of industrial solid waste; however, its utilization rate remains only around 30%. The presence of f-CaO is the main factor in its widespread application. Currently, the carbonation of steel slag is mainly through indirect wet mineralization, which is difficult to implement on an industrial scale. Direct dry carbonation, on the other hand, consumes more energy due to its slow kinetics. In this study, steam coupled with CO_2_ was used to directly mineralize steel slag, a process fully compatible with existing iron and steel industry treatment processes. The required temperature can be achieved using the waste heat from hot steel slag, eliminating the need for additional heat supply. With 15% steam injection, the CaCO_3_ content increased to 12.02 g/100 g (52.8 kg CO_2_ t^−1^ slag utilization), representing a 16.7% improvement. After mineralization, the f-CaO decreased to 0.61%, with 91.73% of f-CaO in steel slag mineralized. The mineralization efficiency of f-CaO increased by 20.24%. This enhancement was attributed to steam entering the interior pores of steel slag, generating intermediate Ca(OH)_2_, causing steel slag particle breakage and fully exposing the previously enclosed f-CaO for complete carbonation. To further utilize flue gas, the effects of different CO_2_ concentrations on carbon fixation were investigated. At a concentration of 20% CO_2_, the carbon fixation reached 69.90% of that achieved at 100% CO_2_. This research not only addresses the stability issues of steel slag but also reduces CO_2_ emissions and effectively utilizes waste heat, making the process suitable for large-scale industrial application.

## 1. Introduction

Steel slag is a by-product of the steel production process and has become one of the largest sources of industrial solid waste, accounting for approximately 10% to 15% of crude steel production [1]. The global annual output of steel slag is about 190–290 million tons, and by 2025, China’s steel slag production will reach about 120 million tons [2]. In recent years, with the development of the steel industry, the output of steel slag has also been increasing rapidly. However, the effective utilization rate of steel slag remains only at about 30% [3]. A large amount of steel slag occupies land, causes soil pollution, and damages the ecological environment. Therefore, it is urgent to improve the utilization of steel slag. The composition of steel slag is similar to that of cement clinker, and it possesses certain hydraulic properties [4]. Concurrently, steel slag exhibits high mechanical strength. Therefore, steel slag can be used as an aggregate, substituting for cement and other building materials [5,6]. This is currently the most widely employed method for disposing of a large quantity of steel slag.

The existence of f-CaO in steel slag leads to poor bulk stability, which remains a significant limiting factor for its resource utilization [7]. This issue can be mitigated through the carbonation of steel slag [8]. The roll crushing−residual heat with the autoclaving process is one of the advanced technologies for steel slag treatment in China. During the process, steel slag is crushed into different particle sizes by rolling. However, this technology requires a substantial amount of water. The addition of water helps to eliminate f-CaO, thereby addressing the poor stability of steel slag [9]. It is also necessary to reduce the temperature of steel slag for safety reasons and to facilitate subsequent processing steps. Unfortunately, this leads to the loss of waste heat from the steel slag. Additionally, during the iron and steel smelting process, a large amount of low-calorific-value flue gas, rich in CO_2_, is generated. This gas exhibits poor combustion efficiency and high treatment costs, and its direct discharge can cause serious environmental pollution [10]. Therefore, using flue gas for the direct mineralization of hot steel slag could not only stabilize f-CaO and improve slag stability, but also effectively utilize the residual heat of the steel slag. Ultimately, this approach could achieve a win–win situation for steel slag stabilization and CO_2_ emission reduction.

Numerous researchers have utilized steel slag for CO_2_ mineral carbonation, with the methodologies mainly divided into direct and indirect approaches [11]. Currently, the carbonation of steel slag is predominantly conducted through indirect wet mineralization, both domestically and internationally. Indirect carbonation generally consists of two stages. Initially, Ca^2+^ and Mg^2+^ ions are extracted from the steel slag using a leaching solution, such as hydrochloric acid, nitric acid, citric acid, or ammonium salts, followed by carbonation in an alkaline environment [12,13,14,15]. Jo et al. [16] employed hydrochloric acid (HCl) and sodium hydroxide (NaOH) to leach Ca^2+^ from steel slag and subsequently synthesized high-purity nano-calcium carbonate (nCaCO_3_), which demonstrated that nCaCO_3_ with a purity of 98.5% could be successfully obtained through CO_2_ carbonation. Although indirect mineral carbonation offers relatively high efficiency, the process is technologically complex and energy-intensive, which significantly limits its scalability and industrial application.

Compared with indirect mineralization, direct mineralization of steel slag using CO_2_ is well-established, straightforward to implement, and suitable for industrial-scale production. In direct mineralization, flue gas with a low calorific value and rich in CO_2_ can be used for the homologous treatment of steel slag, thereby achieving a synergistic treatment of both steel slag and flue gas. However, the kinetics of the direct carbonation reaction are slow, resulting in low conversion rates. Revathy et al. [17] investigated the carbon sequestration capacity of steel slag under ambient conditions for 3 h and observed a carbonation amount of only 11.1 g CO_2_ kg^−1^ slag. Santos et al. [18] explored the carbon sequestration capacity of steel slag under high-temperature and high-pressure conditions (T = 650 °C, P (CO_2_) = 20 bar) and found that the content of CaCO_3_ generated during carbonation ranged from 9% to 26%.

The large-scale implementation of direct carbonation remains challenging due to its low mineralization efficiency. Therefore, it is essential for direct carbonation to improve its carbonation efficiency. Numerous studies have demonstrated that the introduction of steam can significantly enhance the mineralization efficiency of steel slag [19,20]. In the 1970s, Dobner et al. [21]. pioneered the investigation into the carbonation of calcined dolomite in the presence of steam and discovered that steam possessed catalytic properties when facilitating the reaction between CaO and CO_2_, particularly at lower temperatures (below 550 °C). Tamas et al. [22]. showed that the existence of H_2_O refined the particle size distribution and acted as an accelerator for the carbonate, where the output of calcite under wet conditions (0.246 kg CO_2_/kg) was almost three times higher than that under dry conditions (0.083 kg CO_2_/kg).

Symon et al. [23] and Wang et al. [24] thought that the promotion of steam on the carbonation process of steel slag was related to the formation of Ca(OH)_2_. Lu et al. [23] pointed out that the addition of water would force the product volume to be broken, thus affecting the particle morphology and improving the adsorption performance. While Nikulshina et al. [25] indicated that the addition of steam greatly improved the reaction kinetics, with initial reaction speeds 22 times and 9 times higher than the dry carbonization of CaO and Ca(OH)_2_, respectively. However, some studies showed that adding steam contributes little to CO_2_ absorption [18].

Two mutually exclusive hypotheses persist regarding the role of steam in steel slag carbonation. One postulates that steam accelerates reaction kinetics, while the other contends that steam has little contribution in the carbonation process; however, neither conjecture has survived rigorous experimental verification. Based on the above research, this study conducted carbonation experiments and thermodynamic calculations to explore the role of steam in the direct mineralization of CO_2_ using steel slag and expounded upon the mechanism by which steam improves the carbonation efficiency. This research aimed to address the issue of free CaO(f-CaO) in steel slag by integrating water vapor with CO_2_ direct mineralization technology. This approach couples low-grade waste heat, steam, and CO_2_ in iron and steel plants, realizing a “one-step” simultaneous digestion of f-CaO, solidification of CO_2_, and recovery of waste heat without changing the existing slag treatment processes, which opens up a new path with low-carbon and zero additional energy consumption for the large-scale and high-value utilization of steel slag.

## 2. Materials and Methods

### 2.1. Raw Materials

**Figure 1:**
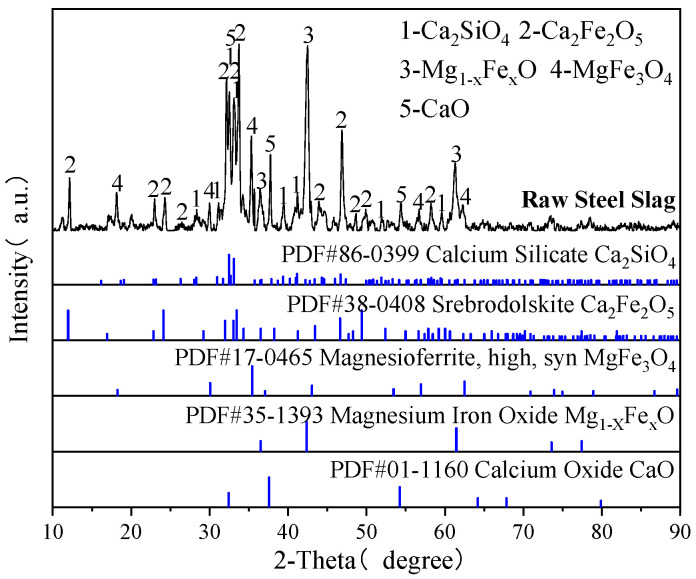
XRD patterns of the raw steel slag.

The steel slag used in the experiment was obtained from Zhanjiang, China. After undergoing 2 h of grinding in a ball mill, the steel slag was sieved into samples with different particle sizes for the carbonation experiment. High-purity N_2_ (99.99%, Beijing Jingcheng Gas Co., Ltd., Beijing, China) was used in the experiment. High-purity CO_2_ (99.95%, Beijing Jingcheng Gas Co., Ltd., Beijing, China) was used in the experiment, while simulated flue gas was prepared by mixing N_2_ and CO_2_ at controlled ratios. The chemical and phase compositions of steel slag determined by X-ray fluorescence (XRF) and X-ray diffraction (XRD) are shown in Table 1 and Figure 1, respectively. According to GB/T 38216.3-2023 [26], the content of f-CaO in steel slag was determined to be 7.34%.

### 2.2. Materials Characterization and Analysis

**Figure 2:**
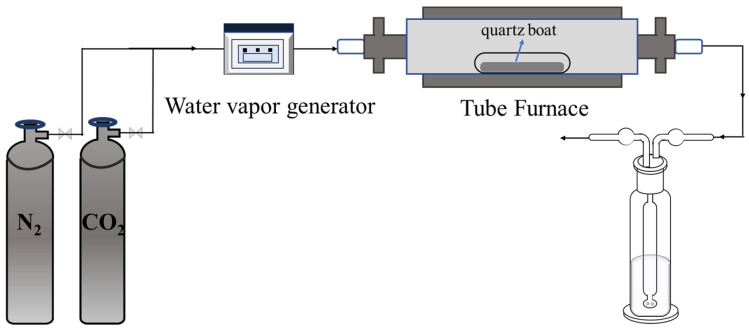
Experimental flow chart.

The chemical compositions of the slags after carbonation were also determined using XRF and XRD. Scanning electron microscopy (SEM) coupled with energy dispersive spectroscopy (EDS) was employed to observe the surface morphology and elemental composition of samples. The operation was conducted under a high vacuum mode with an accelerating voltage of 15 kV. Prior to testing, the samples were coated with a thin film of Au/Pt. The structures of the carbonation products were determined by Fourier transformation infrared spectroscopy (FTIR) within a wavenumber range of 4000~400 cm^−1^. Thermogravimetric–differential scanning calorimetry (TG-DTA) was used to analyze the mass changes in different carbonated steel slag samples, thus quantitatively describing the carbonation effect. The samples were heated from 25 °C to 1000 °C at a rate of 10 °C/min under a constant N_2_ flow of 100 mL/min, with continuous mass measurement throughout the process. In the experiment, the water vapor was quantitatively controlled using a steam generator. The experimental flow chart is shown in Figure 2.

### 2.3. Program for Gas–Solid Direct Carbonation of Steel Slag

The raw steel slag was crushed, ground, and sieved into five different particle size ranges: <75 μm, 75~150 μm, 150~500 μm, 500~1000 μm, and 1000~2000 μm. Approximately 1 g of each steel slag sample was placed in a quartz boat and introduced into a tube furnace for carbonation. During the experiment, N_2_ was used as a protective gas. The tube furnace was heated at a rate of 10 °C/min to the target carbonation temperature, which was typically 450 °C, 500 °C, 550 °C, 600 °C, or 650 °C. In the actual steelmaking process, these temperatures can be achieved during the cooling of hot slag without additional heat supplement. Once the target temperature was reached, the CO_2_ flow rate (30–70 mL/min), CO_2_ concentration (20%, 40%, 60%), and steam content (5%, 10%, 15%, 20%) were adjusted as required. The carbonation reaction was then maintained at the set temperature for 2 h.

### 2.4. Carbonization Efficiency Measurement

The amount of fixed CO_2_ and the corresponding CaCO_3_ formation were calculated using Equations (1) and (2),

(1) $${\mathrm{CO}}_{2}\left[\%\right]=\frac{m_{1073k}-m_{873k}}{m_{o}}\times 100$$

(2) $${\mathrm{CaCO}}_{3}\left[\%\right]=\frac{{\mathrm{CO}}_{2}[\%]\times 100}{44}\times 100$$

where CO_2_[%] was the amount of CO_2_ fixed in the carbonated steel slag (%), CaCO_3_[%] was the CaCO_3_ production (%), m_0_ was the initial mass of the steel slag (mg), m_1073k_ was the mass of steel slag at1073 k (mg), and m_873k_ was the mass of steel slag at 873 k (mg). The temperature range of 873–1073 k corresponds to CaCO_3_ decomposition.

## 3. Results and Discussion

### 3.1. Effect of Different Influencing Factors on Gas–Solid Direct Carbonation Ability of Steel Slag

**Figure 3:**
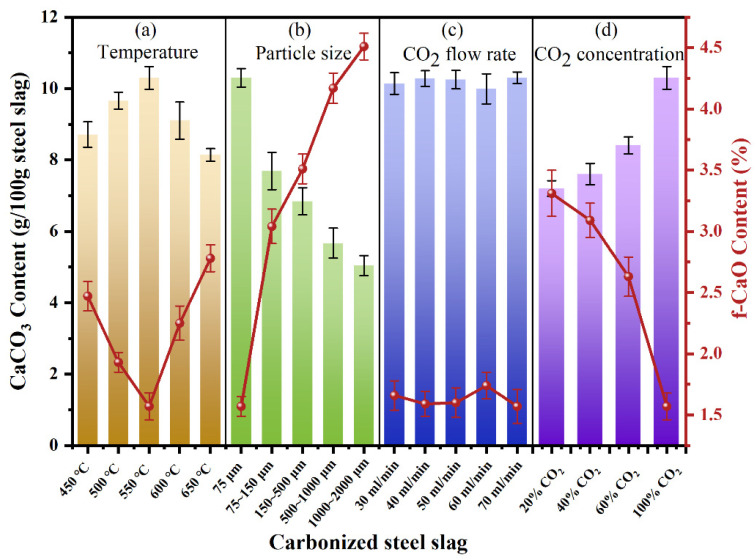
CaCO_3_ and f-CaO content after carbonation of steel slag: (**a**) Temperature, (**b**) particle size, (**c**) CO_2_ flow rate, and (**d**) CO_2_ concentration.

Due to the existence of f-CaO, the stability of steel slag is poor, which greatly limits its application. Gas–solid direct carbonation of steel slag primarily proceeds through the reaction between f-CaO and CO_2_, during which f-CaO is converted into a stable carbonate [27]. As depicted in Figure 3, the amount of CaCO_3_ produced varies significantly with temperature and particle size, indicating a substantial influence of these parameters on carbonation efficiency. In contrast, the CO_2_ flow rate exhibits a relatively minor impact on carbonation efficiency. As illustrated in Figure 3a, with the increase in reaction temperature, the amount of CaCO_3_ first increases and then decreases with higher temperature. The carbonation of steel slag reaches its maximum efficiency at 550 °C, corresponding to 10.30 g CaCO_3_/100 g of steel slag, where the carbonation proceeds slowly due to insufficient reaction kinetics. However, when the temperature reaches a certain value, the exothermic reaction of carbonization inhibits further reaction [28,29]. Figure 3b shows the influence of steel slag particle size on CaCO_3_ production. Smaller particle sizes result in higher carbonation production. When the particle size is below 75 μm, the carbonation effect reaches its maximum This improvement arises because smaller particles expose more f-CaO on their surfaces, enhancing the reaction rate and overall carbonation degree. Figure 3c reveals the negligible influence of CO_2_ flow rate on steel slag carbonation, indicating that 30 mL/min was sufficient for the experimental slag quantity. To sum up, for a gas–solid reaction, increasing temperature and reducing particle size can accelerate the carbonation reaction. When the temperature was 550 °C, the particle size was 75 μm, and the CO_2_ flow rate was 30 mL/min, the maximum amount of CaCO_3_ was 10.3 g/100 g steel slag. After direct gas–solid carbonation, the residual f-CaO content in steel slag was 1.57%, indicating that 76.29% of f-CaO in steel slag was mineralized.

To assess the feasibility of utilizing CO_2_-rich flue gas for direct carbonation, Figure 3d further shows the influence of different CO_2_ concentrations on steel slag carbonation. When the concentration of CO_2_ is 20%, the carbonation efficiency remains relatively high, producing 7.20 g CaCO_3_/100 g steel slag, equivalent to 69.90% of the efficiency under pure CO_2_, which confirms the feasibility of using flue gas for steel slag mineralization.

**Figure 4:**
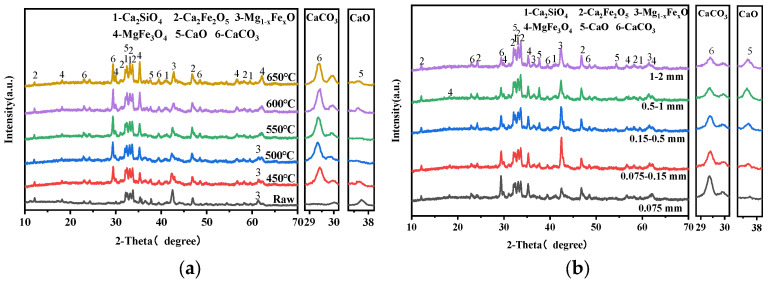

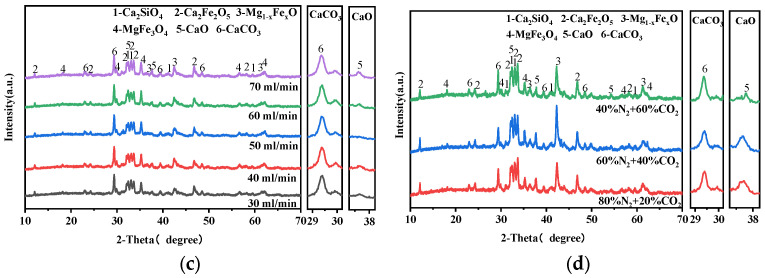
XRD patterns of carbonated steel slag: (**a**) Temperature, (**b**) particle size, (**c**) CO_2_ flow rate, and (**d**) CO_2_ concentration.

Figure 4 presents the XRD patterns of carbonated steel slag under various carbonization conditions. All carbonated steel slag samples exhibit a typical calcite diffraction peak at 2θ = 29.40°, while the intensity of f-CaO peaks decreases significantly after carbonation. Changes in carbonation temperature and particle size of steel slag cause obvious variations in phase compositions, whereas the CO_2_ flow rate produces negligible changes in CaCO_3_ and CaO peak intensities, which is consistent with CaCO_3_ production in Figure 3. When the CO_2_ concentration is 20% (Figure 4d), the characteristic peak intensity of CaCO_3_ in the carbonized steel slag still remains distinct. In addition, the peak intensity of dicalcium–silicate- and iron-containing minerals in carbonated steel slag samples remain essentially unchanged, indicating that only f-CaO is involved in the gas–solid carbonation reaction.

**Figure 5:**
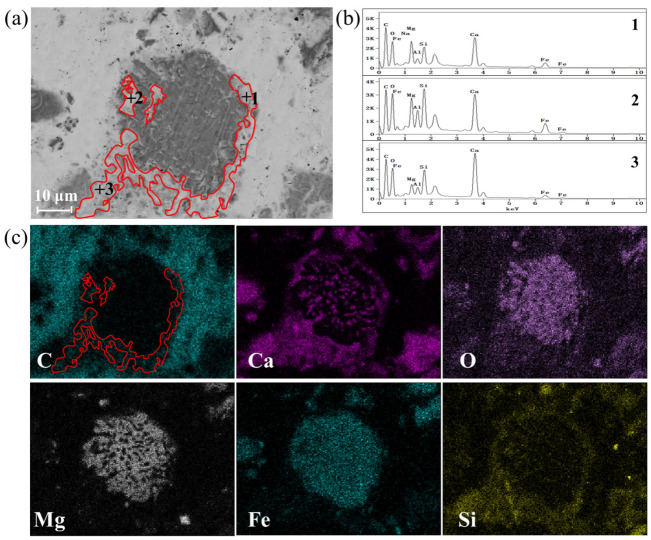
BSE image and element distribution of gas–solid carbonated steel slag particles: (**a**) SEM of carbonated steel slag, (**b**) EDS result of CaCO_3_ formation at point 1~3, and (**c**) mapping of carbonated steel slag.

Figure 5 shows the SEM and BSE results to further confirm the carbonation behavior. Steel slag appears in the form of larger particles, while CaCO_3_ produced by carbonation shows a smaller particle size. The elements of Ca, Fe, Mg, and O are mainly concentrated in the central area of the particles, suggesting the presence of phases such as Mg_1−x_Fe_x_O and Ca_2_Fe_2_O_5_. In addition, Ca and C also gathers loosely around the particles, indicating that small pieces of CaCO_3_ were formed and distributed around the surfaces of steel slag particles, forming a CaCO_3_ layer. It can be observed from Figure 5 that CaCO_3_ is mainly distributed around the steel slag particles and forms a CaCO_3_ layer.

For gas–solid carbonation, the carbonation process occurs initially on the surface of steel slag particles. The steel slag after carbonation still presents relatively complete particles. As the reaction progresses, a CaCO_3_ layer forms on the surfaces of steel slag particles, creating a protective product coating, which prevents further effective contact between CO_2_ gas and the unreacted part of steel slag. It is difficult for CO_2_ to diffuse into the steel slag, which inhibits its further carbonization.

### 3.2. Effect of Steam on Gas–Solid Direct Carbonation Performance of Steel Slag

Introducing steam into gas–solid carbonation significantly enhances the carbon fixation of steel slag [19,20].

**Figure 6:**
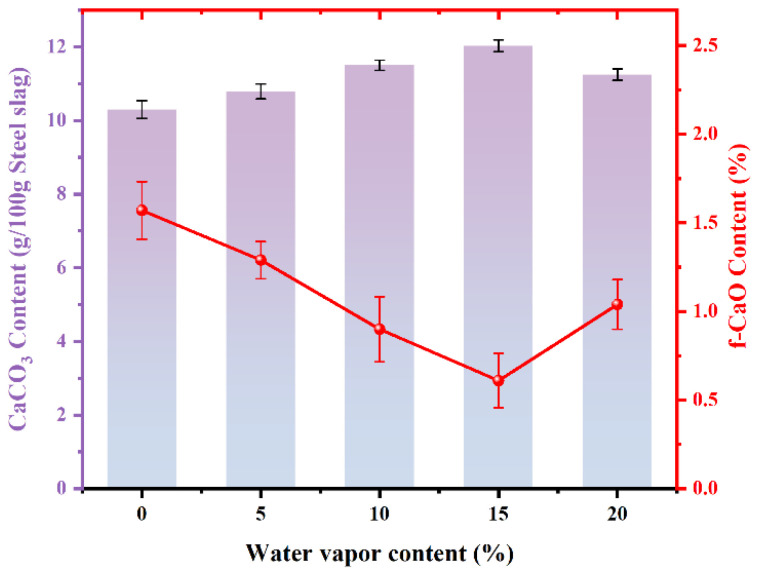
Effect of steam on carbonation of steel slag CaCO_3_ and f-CaO production.

Figure 6 shows the amount of CaCO_3_ produced by carbonated steel slag with different steam contents. The content of carbonized CaCO_3_ initially increases and then decreases with rising steam content. The amount of CaCO_3_ reaches 12.02 g/100 g steel slag upon the injection of 15% steam, which is an increase of 16.70% when compared to its content under pure CO_2_. Correspondingly, the residual f-CaO content decreases sharply by 0.61%, indicating that 91.73% of the initial f-CaO has been mineralized. Compared with the mineralization process without steam injection, the mineralization efficiency of f-CaO has increased by 20.24%, suggesting that the injection of steam promoted the almost complete reaction of f-CaO. However, excessive steam inhibits carbonation because thick water films form on particle surfaces, which prevents CO_2_ entry and restricts the reaction to the outer surface layer [30]. Table 2 lists the performance comparison between the results of this study and others, in reference to the strengthening effects of steam in the direct carbonization of steel slag. In this study, the carbonation efficiency and the residual amount of f-CaO in steel slag are better than those reported in the references.

**Figure 7:**
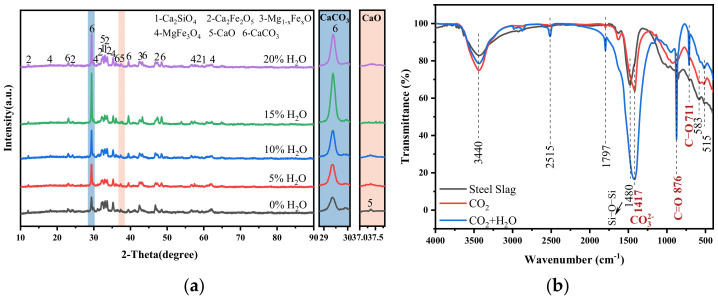
Effect of steam on X-ray diffraction (XRD) and Fourier transformation infrared spectroscopy (FTIR) of carbonated steel slag. (**a**) XRD diagram of carbonated steel slag with different steam content; (**b**) FTIR analysis of steel slag before and after carbonation.

Figure 7a shows the XRD patterns of carbonated steel slag with various steam contents. The characteristic calcite peak intensity reaches the maximum at 15% steam, while the f-CaO peak intensity nearly disappears. Other phases remain unchanged, confirming that f-CaO is the main phase involved in the reaction. Compared with pure CO_2_ gas carbonation (Figure 4), the peak intensity of calcite is significantly stronger in the presence of steam.

Figure 7b displays the FTIR spectra of steel slag before and after carbonization. For the original steel slag sample, silicate minerals in steel slag, such as dicalcium silicate and tricalcium silicate, have characteristic absorption peaks around 1480 cm^−1^, corresponding to the tensile and bending vibration of Si-O-Si [35]. The absorption band of carbonated steel slag around 711 cm^−1^ can be ascribed to the vibration of the C-O bond in calcite. The band at 876 cm^−1^ represents the out-of-plane bending of the C=O bond in calcite. The absorption peaks around 1417 cm^−1^, caused by carbonate, are usually related to the symmetric stretching vibration mode of the carbonate ion (CO_3_^2−^) in calcium carbonate [36,37]. It is clearly observed that when steel slag was injected with steam, the signal intensities of the absorption peaks at 876 cm^−1^ and 1417 cm^−1^ increase obviously. Meanwhile, in the FTIR diagrams of carbonized steel slag injected with steam, absorption peaks of calcite near 1797 cm^−1^ and 2515 cm^−1^ appear, indicating that the presence of steam promotes the carbonization process of steel slag [38,39]. This observation is consistent with the XRD results (Figure 7a) and CaCO_3_ production (Figure 6) of carbonated steel slag after adding steam, suggesting that the increased strength of absorption peaks in the FTIR spectra and the changes in XRD patterns, along with the increased CaCO_3_ production, support the conclusion that steam enhanced the carbonization process.

**Figure 8:**
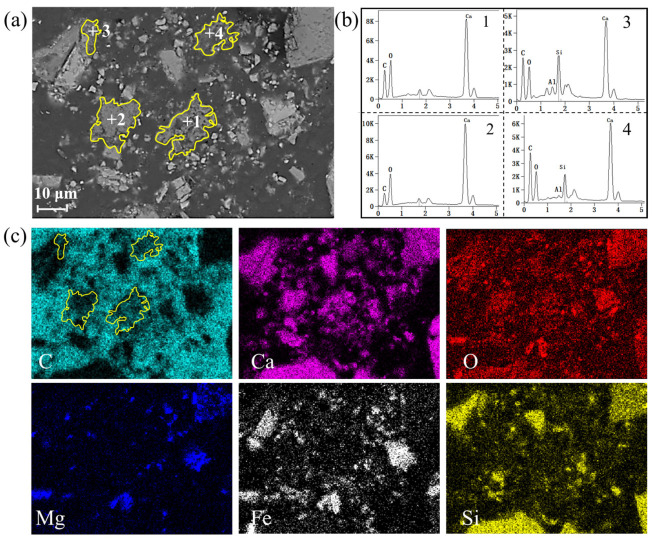
Effect of steam on microstructure of carbonated steel slag: (**a**) SEM of carbonated steel slag, (**b**) EDS result of CaCO_3_ formation at point 1~4 (The point 1~4 respectively represent +1~+4 in Figure 8a), and (**c**) mapping of carbonated steel slag.

Figure 8 shows the BSE images and corresponding element distribution of carbonated steel slag after adding steam. It is clearly observed that the carbon steel slag particles are obviously broken after steam treatment. Compared with pure CO_2_ carbonation (Figure 8), the sample with steam exhibits smaller particle size, and a more dispersed distribution of CaCO_3_. Under the steam/CO_2_ mixed gas atmosphere, the generated CaCO_3_ is more dispersed throughout the slag matrix as small particles (Point 1~4 in Figure 8). Overall, the addition of steam can pulverize the particles of steel slag, increase the contact area between CO_2_ and steel slag, and consequently promote the generation of CaCO_3_.

### 3.3. Carbonation Mechanism by Steam

**Figure 9:**
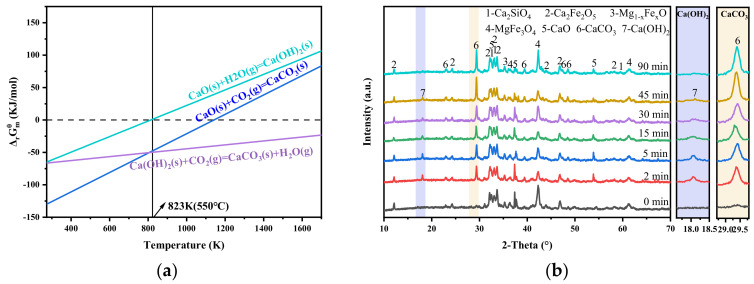
Effect of adding steam on gas–solid carbonation of steel slag. (**a**) Effect of steam on Gibbs free energy of CaO; (**b**) XRD diagram of carbonated steel slag after adding steam at different time periods.

The above experiments demonstrate that steam has a positive effect on the carbonation process of steel slag. Based on the thermodynamic principle, the influence of steam on the carbonation of steel slag was analyzed in the standard state. Figure 9a shows the change in Gibbs free energy of reactants under the condition of a steam/CO_2_ atmosphere. As illustrated in Figure 9a and summarized in Table 3, Equations (3)–(5) can proceed spontaneously within the temperature range of 273 to 814 K, under standard conditions. At 550 °C (823 K), the reactivities of Equations (3) and (5) are comparable and both more favorable than that of Equation (4). Therefore, when the reactive gas contacts the steel slag, CO_2_ preferentially reacts with f-CaO exposed on the surface of steel slag (Equation (3)). At the same time, steam permeates the porous slag structure and reacts with f-CaO to generate Ca(OH)_2_ (reaction 4). The formation of Ca(OH)_2_ involves a volume expansion of about 98% [40], which pulverizes the steel slag and exposes the f-CaO inside the steel slag. The newly exposed f-CaO subsequently reacts with CO_2_, while Ca(OH)_2_ is further carbonated according to reaction (5). Therefore, the presence of steam enables a more complete conversion of f-CaO in steel slag.

To verify this conjecture, Figure 9b analyses the phase evolution of carbonized steel slag at different time periods in the presence of steam. During the process of steel slag carbonization, the extension of carbonization time has a significant effect on the phase composition of steel slag. At the initial stage, the characteristic peaks of CaCO_3_ and Ca(OH)_2_ appear simultaneously, while the peak intensity of f-CaO weakens, indicating simultaneous formation of CaCO_3_ and Ca(OH)_2_ upon gas–slag contact. With increasing carbonization time, the peak intensity of CaCO_3_ increases, whereas the peak intensities of Ca(OH)_2_ and f-CaO gradually decrease, suggesting progressive conversion of f-CaO and Ca(OH)_2_ into CaCO_3_. After 90 min, the characteristic peak of Ca(OH)_2_ disappears, indicating that all Ca(OH)_2_ has been converted into CaCO_3_. With the introduction of steam, the content of f-CaO in the mineralized steel slag decreases to 0.61% (Figure 6) compared to that of 1.57% without steam injection, demonstrating a 20.24% improvement in the mineralization efficiency.

**Figure 10:**
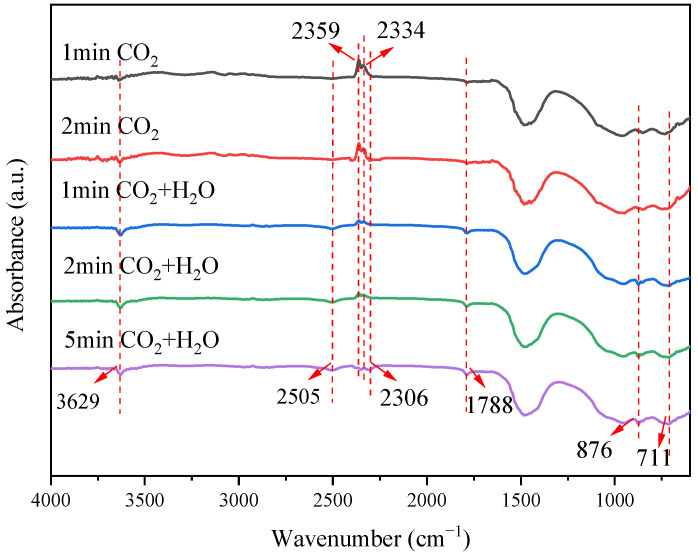
In situ FTIR spectra of steel slag in CO_2_ and CO_2_/H_2_O atmosphere.

The above experimental results strongly support the positive promotion of steam on the carbonation process of steel slag. The carbonation of steel slag is obviously accelerated by the formation of the intermediate of Ca(OH)_2_. To further confirm the role of steam, this dynamic process was clearly conducted by in situ FTIR. Figure 10 shows the in situ FTIR spectra of steel slag in CO_2_ and CO_2_ + H_2_O atmospheres at 550 °C. The doublets at 2359 and 2334 cm^−1^ correspond to the ν_3_ asymmetric stretching vibration of gaseous CO_2_ split by Fermi resonance. A new band at 2306 cm^−1^, appearing only in the CO_2_ + H_2_O atmosphere, can be attributed to linearly adsorbed CO_2_ species on basic sites, implying that steam enhances CO_2_ chemisorption, possibly via surface HCO_3_^−^. The bands at 1788, 876, and 711 cm^−1^ are assigned to the ν_2_ bending, ν_4_ in-plane bending, and ν_2_ lattice modes of CO_3_^2−^, respectively, indicating the presence of calcitic CaCO_3_. Their integrated intensities increase monotonically over time and are significantly larger in the presence of steam, confirming that H_2_O accelerates CaCO_3_ formation. These observations support the sequential pathway CaO → Ca(OH)_2_ → CaCO_3_, which proceeds faster than direct gas–solid carbonation. The 3629 cm^−1^ peak, assigned to surface -OH groups and possibly including transient Ca(OH)_2_ stabilized on the slag matrix, intensifies within the first minute under CO_2_ + H_2_O, indicating rapid water adsorption and hydroxylation of the slag surface.

To sum up, in a pure CO_2_ atmosphere, the characteristic peak of carbonate increases slowly, because the reaction rate is limited by the direct carbonation of surface-active CaO sites. After the introduction of steam, H_2_O significantly enhances the carbonation rate and the adsorption capacity of CO_2_ through the formation of the Ca(OH)_2_ intermediate.

**Figure 11:**
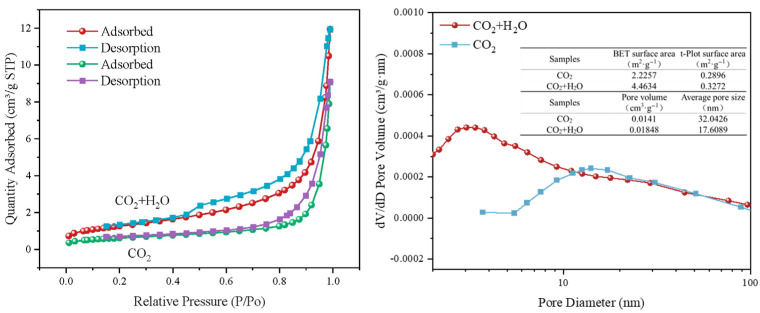
BET diagram of mineralized steel slag before and after steam injection.

Figure 11 presents the N_2_ adsorption–desorption isotherms and pore structure parameters of steel slag carbonated at 550 °C for 2 h under CO_2_ and CO_2_ + H_2_O atmospheres. Compared to conditions under pure CO_2_, steam introduction increases the BET surface area from 2.23 m^2^·g^−1^ to 4.46 m^2^·g^−1^, indicating enhanced activation of Ca/Mg bearing phases and the creation of micro- and mesopores. The mean pore size decreases from 32 nm to 17 nm, while the pore volume increases by 31% to 0.0185 cm^3^·g^−1^. The transition from “fewer, larger” to “numerous, medium sized” pores facilitates CO_2_ diffusion.

The steep uptake at P/P_0_ < 0.1 and the hysteresis loop (type H_3_/H_4_) imply a higher micropore fraction and slit shaped pores formed by the stacking of lamellar particles. Under pure CO_2_, the predominance of macropores and low pore volume suggest partial blockage by nascent carbonates. In the presence of steam, transient Ca(OH)_2_-like surface hydroxyls are formed and subsequently carbonated to CaCO_3_, reopening and enlarging the pore network.

Consequently, steam not only increases the number of reactive sites but also generates a more open and accessible pore structure, thereby enhancing the gas–solid carbonation efficiency of steel slag.

**Figure 12:**
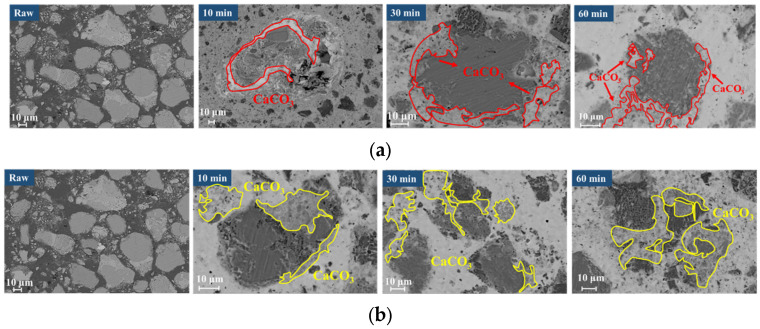
Microscopic morphology of carbonated steel slag. (**a**) Gas–solid carbonation; (**b**) Gas–solid carbonation with added steam.

Figure 12 shows the BSE images of carbonated steel slag at different time periods in the presence of steam.

In the pure CO_2_ atmosphere (Figure 12a), steel slag particles remain at larger particle sizes and relatively intact morphologies after 60 min of reaction. With the gradual generation of CaCO_3_, unreacted regions became coated with a dense CaCO_3_ layer, confining the reaction to surface regions.

In contrast, under the steam/CO_2_ mixed atmosphere (Figure 12b), combined with the analysis in Figure 9, the f-CaO in the steel slag reacts with steam to generate Ca(OH)_2_, accompanied by a significant volume-expansion effect, leading to the effective crushing of steel slag particles into smaller sizes, as clearly observed in Figure 12b. Compared with the particles in Figure 12a, those in Figure 12b exhibit a significantly reduced size. The crushing of steel slag particles exposes the f-CaO within the steel slag, which greatly improves further contact between f-CaO and CO_2_, and then promotes the generation of CaCO_3_, resulting in a denser distribution of CaCO_3_ in the mixed atmosphere of steam/CO_2_. These microstructural changes align well with the experimental results of CaCO_3_ content in the presence of steam (Figure 6) and the BET results (Figure 7), providing intuitive evidence for the positive role of steam in promoting carbonation of steel slag.

**Figure 13:**
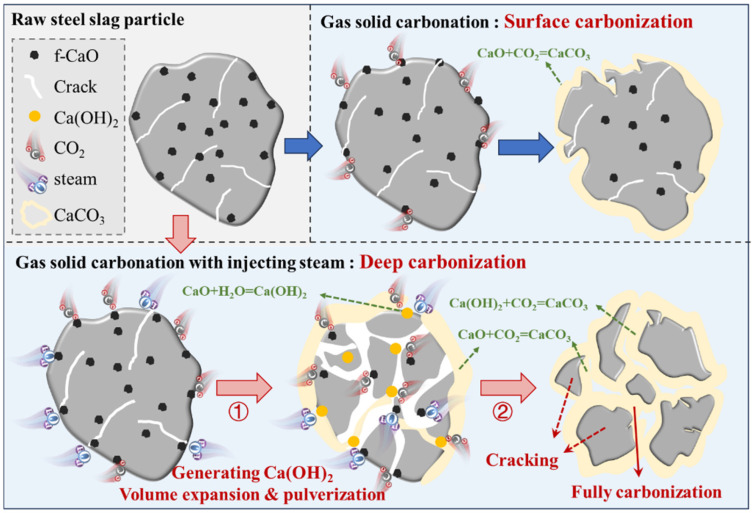
Diagram of steam carbonation mechanism.

Based on the above thermodynamic calculation and gas–solid carbonation experimental results, a schematic diagram of the steam-assisted carbonation mechanism of steel slag is proposed in Figure 13.

In a pure CO_2_ atmosphere, the formation of CaCO_3_ is primarily due to the direct reaction between f-CaO and CO_2_ on the surface of steel slag. As the reaction progresses, a CaCO_3_ layer eventually encapsulates the unreacted steel slag, limiting further carbonation reactions.

When steam was introduced, f-CaO on the surface of steel slag initially reacted with CO_2_ for carbonation. Simultaneously, steam permeates into the steel slag through its surface pores and reacted with f-CaO to generate Ca(OH)_2_. This process was accompanied by a significant volume expansion, leading to the pulverization of steel slag into smaller particles and a substantial increase in the specific surface area of the steel slag. This volume expansion and pulverization exposed the previously enclosed f-CaO within the steel slag to the surface, further increasing the contact area between f-CaO and CO_2_, thereby promoting the generation of CaCO_3_. The Ca(OH)_2_ formed by the reaction between steam and f-CaO in steel slag can be further carbonized by CO_2_ to produce CaCO_3_. The presence of steam significantly improves the process by promoting the digestion of free CaO (f-CaO) in steel slag and thereby enhancing carbonation efficiency.

To sum up, steam promotes the digestion of f-CaO and improves carbonation efficiency through the volume-expansion effect, which significantly advances the carbonation process of steel slag by increasing the surface area available for reaction and facilitating the exposure of internal f-CaO to CO_2_. Moreover, the process is compatible with existing technological conditions in the iron and steel industry, utilizing the residual heat of hot steel slag without additional energy input. Consequently, this process simultaneously stabilizes steel slag, reduces CO_2_ emissions, and enables large-scale industrial application.

## 4. Conclusions

In this research, steam was used to directly enhance mineralization of steel slag. The effects of CO_2_ flow rate, CO_2_ concentration, and steam addition on gas–solid carbonation of steel slag were discussed, and the strengthening mechanism of steam on steel slag carbonation was expounded.

1. Under the condition of CO_2_ atmosphere, the carbonation effect of steel slag exhibits optimal performance at 550 °C with a particle size of 75 μm and a CO_2_ flow rate of 70 mL/min. The amount of CaCO_3_ produced reaches 10.3 g/100 g of steel slag. After direct gas–solid carbonation, the f-CaO content in steel slag decreases to 1.57%, indicating that approximately 76.29% of f-CaO in steel slag is mineralized. When the CO_2_ concentration is reduced to 20%, the carbon fixation remains at 7.60 g CaCO_3_/100 g steel slag, equivalent to 73.79% of that obtained with pure CO_2._
2. Under a steam/CO_2_ atmosphere, when the addition amount of steam is 15%, the carbonation of steel slag reaches its maximum efficiency. The amount of CaCO_3_ produced reaches 12.02 g/100 g steel slag, which is 16.7% higher than that achieved in the pure CO_2_ atmosphere. After mineralization, the f-CaO content decreases to 0.61%, corresponding to a 91.73% mineralization efficiency. Overall, the f-CaO conversion increases by 20.24% compared with carbonation without steam.
3. The addition of steam significantly promotes the activation of f-CaO and leads to the formation of additional micro/mesoporous structures. The average pore size decreases from 32 nm to 17 nm, while the pore volume increases from 0.0141 cm^3^·g^−1^ to 0.0185 cm^3^·g^−1^, representing an increase of 31%, indicating a transition of the pore structure from “few in number and large in scale” to “many in number and medium in scale”. The presence of steam enhances both the carbonation rate and CO_2_ adsorption capacity by generating the Ca(OH)_2_ intermediate.
4. The mechanism of direct mineralization of steel slag enhanced by steam was expounded. Steam enters the open pores of the slag and reacts with f-CaO to generate Ca(OH)_2_, leading to volume expansion and consequently causing physical crushing and refinement of steel slag particles. The volume expansion and pulverization caused the f-CaO to be exposed to the surface, facilitating an increased contact between the f-CaO and CO_2_. Meanwhile, the newly generated Ca(OH)_2_ further reacts with CO_2_ to produce CaCO_3_, thereby accelerating the entire carbonation reaction.

This study successfully achieved the dual goals of solving the stability problem and reducing CO_2_ emissions simultaneously. Moreover, the waste heat generated during the steel slag production process is effectively utilized, improving overall energy efficiency. The process maintains production stability without requiring new equipment, thereby avoiding additional costs and technical risks. Hence, the proposed process is compatible with the existing steel slag treatment process and can be directly applied on a large scale without changing the existing process flow.

However, certain limitations remain in this study. In the gas–solid carbonation system, the promoting effect of steam primarily targeted f-CaO, thereby restricting the theoretical carbon fixation capacity to the f-CaO content. In the follow-up study, high calcium/magnesium-based industrial by-products can be introduced through the multi-element solid waste synergistic mineralization strategy to expand the carbonation reaction site, thereby achieving a significant improvement in overall carbon sequestration and the co-treatment of multi-solid waste. Furthermore, although this study proposes a new strategy for low-carbon utilization of steel slag, its large-scale application will require systematic investigations in reactor amplification, working condition optimization, and life cycle assessments.

## Figures and Tables

**Table 1:** The chemical composition of the raw steel slag.

Components	CaO	Fe_2_O_3_	SiO_2_	MgO	Al_2_O_3_	MnO	P_2_O_5_	TiO_2_	Cr_2_O_3_	V_2_O_5_	SO_3_	Else	f-CaO *
Content (*wt*%)	42.10	26.30	10.91	6.86	5.25	4.07	2.26	0.82	0.42	0.27	0.26	0.48	7.34

* The f-CaO in steel slag was determined according to GBT 38216.3-2023 [26].

**Table 2:** Comparison of properties of steam promoting direct carbonation of steel slag.

Raw Material	Method	Carbonation Parameter	Carbonation Effect	Ref.
steel slag	direct gas–solid carbonation	H_2_O = 20 mL/min; CO_2_ 40 mL/min; t = 30 min; particle size = 75 μm	calcium carbonate production = 1.38%	[31]
steel slag	direct gas–solid carbonation	T = 60 °C; L/S = 30:1; t = 10 h	carbonation efficiency = 58.64%	[32]
steel slag	direct gas–solid carbonation	T = 70 °C; H_2_O = 20 mL/min; CO_2_ = 80 mL/min	f-CaO residue = 0.91%; conversion rate = 54.95%	[33]
steel slag	direct gas–solid carbonation	composite gas of CO_2_ and water vapor	f-CaO residue = 0.85%	[34]
steel slag	direct gas–solid carbonation	T = 550 °C; H_2_O = 15 mL/min; CO_2_ = 85 mL/min; t = 2 h; particle size = 75 μm	CaCO_3_ fixation capacity = 12.02%; f-CaO residue = 0.61%; conversion rate = 91.73%	This work

**Table 3:** Equation and thermodynamic calculation of aqueous carbonation reaction.

Chemical Reaction Equation	Variation in Standard Gibbs Free Energy of Reaction (kJ/mol)
CaO + CO_2_ = CaCO_3_ (3)	$\Delta_{r}G^{\theta }=-170.933+0.149T$
CaO + H_2_O = Ca(OH)_2_ (4)	$\Delta_{r}G^{\theta }=-97.633+0.120T$
Ca(OH)_2_ + CO_2_ = CaCO_3_+H_2_O (5)	$\Delta_{r}G^{\theta }=-101.039+0.149T$

## Data Availability

The original contributions presented in this study are included in the article. Further inquiries can be directed to the corresponding authors.
